# Single-shot quantitative phase-fluorescence imaging using cross-grating wavefront microscopy

**DOI:** 10.1038/s41598-024-52510-9

**Published:** 2024-01-25

**Authors:** Baptiste Marthy, Maëlle Bénéfice, Guillaume Baffou

**Affiliations:** Institut Fresnel, CNRS, Aix Marseille Univ, Centrale Med, Marseille, France

**Keywords:** Interference microscopy, Cellular imaging

## Abstract

The article introduces an optical microscopy technique capable of simultaneously acquiring quantitative fluorescence and phase (or equivalently wavefront) images with a single camera sensor, avoiding any delay between both images, or registration of images acquired separately. The method is based on the use of a 2-dimensional diffraction grating (aka cross-grating) positioned at a millimeter distance from a 2-color camera. Fluorescence and wavefront images are extracted from the two color channels of the camera, and retrieved by image demodulation. The applicability of the method is illustrated on various samples, namely fluorescent micro-beads, bacteria and mammalian cells.

Fluorescence microscopy is a ubiquitous characterization technique in cell biology^1–3^. Fluorescent labelling of living cells allows one not only to specifically highlight biomolecules, organelles or cellular compartments, but also to map physico-chemical quantities, e.g., ion concentration, action potentials, pH, orientation of molecules, etc. Over the last two decades, fluorescence microscopy has undergone profound improvements, and the development of numerous variants, pushing the limits of imaging in terms of spatial resolution, speed, signal-to-noise ratio, specificity, labelling techniques, and 3D imaging.

Nevertheless, fluorescence microscopy suffers from limitations. It remains invasive by nature, because it requires the labelling of the sample with molecular dyes or proteins^4^. Moreover, live observation cannot be conducted for arbitrarily long periods of time due to photobleaching of the fluorescent tags and photo-toxicity. Finally, fluorescent molecules do not always faithfully tag what they are supposed to, and artifacts can sometimes occur^5^.

Quantitative phase microscopy (QPM) is another imaging technique family dedicated to the field of cell biology^6,7^. Unlike fluorescence microscopy, QPM techniques are label-free and non-specific. They are simply sensitive to the refractive index of the sample. Their main benefit is to provide a much better contrast compared with bright field microscopy. Moreover, the close relation that exists between refractive index and mass density of biological media provides QPM with the unique ability to measure and map the mass of cells in culture, enabling the quantitative monitoring of cell growth, and mass transport at the sub-cellular level^8–11^. Because QPMs are label-free, they do not suffer from any of the above-mentioned drawbacks related to fluorescence microscopy. In particular, there is no photobleaching of any molecular probe, and phototoxicity can be cancelled if using red or infrared illumination, enabling image acquisition for arbitrarily long periods of time, in a non-invasive manner^12^. However, QPMs are not specific by nature. One cannot choose what feature of the cell to highlight, although some recent works involving machine learning attempted to lift this limitation^13,14^.

Fluorescence microscopy and QPM appear thus as complementary approaches, and combining them offers multiple benefits. OPD images show details that cannot be seen in fluorescence images, and vice versa. OPD reveals everything in the cell, without selection, in particular the parts of the cells that are not fluorescently labelled. For instance, it can clearly highlight the lamellipodium, the nucleus, vesicles or mitochondria. On the contrary, fluorescence benefits from specificity, as it highlights only what is labelled in the cell, in particular, objects with too low contrast to be seen on OPD images.

Nevertheless, fluorescence microscopy and QPM are rarely associated. Yet, coupling fluorescence microscopy with a QPM technique could have at least three important applications : (i) it would provide the spatial distribution of biomolecules or organelles (e.g. microtubules, actin, mitochondria, etc.) or physico-chemical parameters, in correlation with the overall morphology of the cell with an excellent contrast, including faint parts of the cells such as the lamellipodium. We envision important application for example in intracellular trafficking studies; (ii) it would enable the correlation between fluorescence information and mass transport within cells; (iii) it could yield the creation of a ground truth database to feed deep learning algorithms aiming at retrieving specific information from QPM images, such as the localization of mitochondria, the state of bacteria (live or dead), etc.

The rare demonstration of fluorescence-phase correlation microscopy stems from the difficulty to combine fluorescence and QPM microscopes. QPMs often involve interferometric measurements and require a dedicated set up. For instance, digital holographic microscopy (DHM), a common QPM technique, cannot be straightforwardly implemented on a pre-existing microscope^15^. The community usually utilizes commercial solutions that are not compatible with fluorescence microscopy. There exists a commercial solution for phase-fluorescence correlation microscopy (fluorescence module, Lyncée tec), coupling DHM with wide-field fluorescence microscopy, but this solution is expensive and bulky. More accessible solutions exist, e.g., consisting of switching the detected wavelength window by the motion of a mechanical element^16^. However, this solution is not suited for the study of fast dynamics, because it implies a delay between the fluorescence and phase images. Using two cameras in parallel with a beam splitter is also possible^17–22^. However, they require the use of an algorithm for the super-imposition of the acquired images, a process called registration. Color cameras have already been associated with QPM techniques. However, it was to demonstrate multispectral QPM, not dual-mode phase-fluorescence correlation imaging^23^. In 2020, Tayal et al.^24,25^ reported a microscopy setup capable of simultaneously measuring fluorescence and phase, with the same camera sensor. However, the setup was based on a Michelson interferometer configuration, which is not a standard equipment in biology labs, and which is highly sensitive to external perturbations (mechanical drift, air flow, etc). Moreover, it requires working in reflection, and does not enable the use of high-NA, oil-immersion objectives.

In this article, we present a simple, cost-effective and sensitive experimental solution for simultaneous phase-fluorescence microscopy, using a single camera acquisition, and without registration. The experimental device involves a 2-color camera designed for this purpose, and a 2D-diffraction grating, associated with each other as a stand-alone device that can be plugged in any pre-existing optical microscope. The first part of the article describes the experimental configuration and the second part illustrates the capabilities of this technique and quantifies its performances by imaging various systems, namely fluorescent beads, bacteria and mammalian cells. For the sake of comprehensiveness, different issues are discussed, such as signal to noise ratio, sensitivity, spatial resolution and chromatic aberrations.

## Results

### Experimental setup

**Figure 1:**
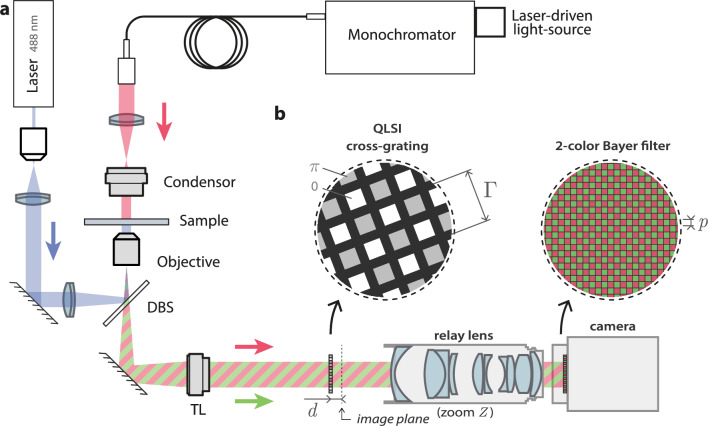
Cross-grating wavefront microscopy (CGM) setup. (**a**) Microscope setup composed of a monochromator (set at 680 nm, bandwidth 6 nm) coupled with a Köhler illumination, a laser illumination (488 nm), a dichroic beam splitter (DBS), and tube lens (TL). (**b**) color-QLSI system, composed of a cross-grating (pitch $\Gamma$), a relay-lens and a 2-color camera (dexel size *p*). The insets represent zooms on the custom-made cross-grating and Bayer filter of the color camera.

Quadriwave lateral shearing interferometry (QLSI) is a wavefront imaging technique based on the use of a 2D-grating (aka cross-grating), placed at a millimeter distance from a camera sensor^26–29^. The cross-grating must be special, composed of black lines defining transparent square holes imprinting a phase shift of 0 or $\pi$ to the light beam according to a checkerboard pattern (Fig. 1b). The raw image, called an interferogram, created on the camera by the QLSI cross-grating is processed to retrieve both the intensity and wavefront profiles of the light beam. Invented^26^ and patented^27^ in 2000 by Primot et al. for applications in laser beam and optical component metrology, it was demonstrated in 2009^28^ that QLSI could also be implemented on an optical microscope, a modality that we recently propose to refer to as cross-grating wavefront microscopy (CGM)^29–31^ (Fig. 1a). Due to its high image definition, CGM could do what other wavefront imaging techniques never achieved: challenging the many quantitative phase microscopies (QPMs) used in biology. Indeed, phase $\varphi$ and wavefront *W* of a light wave are simply proportional, according to the relation

1 $$\begin{aligned} \varphi = \frac{2\pi }{\lambda }W. \end{aligned}$$

Compared with QPM techniques, such as digital holography microscopy (DHM), the main benefits of CGM are its simplicity and its stability. One just needs to plug a camera-like system in the port of a microscope, and the technique is not sensitive to mechanical drift, air flow, vibrations or temperature variations because the interferometry occurs over the protected millimeter distance between the grating and the camera, and does not require a separated reference arm. Moreover, CGM directly measures the quantity of interest in biology: the optical path difference (OPD) $\delta \ell =W$, defined by the optical path variation created by the imaged object once placed in the field of view of the microscope.

In this work, we introduce the use of a color-camera to perform wavefront measurements in parallel with fluorescence measurements. More precisely, we associated a custom-made 2-color camera (CMS4, SILIOS technologies, dexel^32^ size $p=5.5$ µm, 10 bits) with a custom-made QLSI grating ($\Gamma =39$ µm) separated by a relay lens that does not yield monochromatic aberrations or image distortion (VZM 300 Edmund optics, adjustable magnification set to $1.193\times$) (Fig. 1b). The system is set so that the relay-lens creates an image of the grating at the desired distance from the camera sensor. The cross-grating was rotated by around $30 ^\circ$ around the optical axis to avoid Moiré effects (the precise value of the rotation is not important).

Another option would have been to directly implement the QLSI grating in front of the camera, without relay lens, leading to a more compact system. However, implementing a grating at a millimeter distance from a camera sensor is not straightforward as it usually implies to open the atmosphere-controlled chamber of the sensor that is meant to avoid ambient water crystallization on the sensor upon cooling the sensor below $0 ^\circ$C. Moreover, such a solution would prevent from adjusting the grating-camera distance, which is a parameter we wanted to vary in the scope of this article. This is why we implemented a relay-lens between the camera and a separated cross-grating.

The presence of the cross-grating adds dark fringes on the image recorded by the camera. The pitch of the cross-grating was set sufficiently large so that it can be properly sampled by the dexels of the camera according to the Nyquist criterion. In practice, the commonly used criterion in CGM is to have 6 dexels (pixels of the camera) per unit cell of the grating: $\Gamma = 6p^{\prime}$, i.e., $\zeta =3$ dexels per fringe of the interferogram, where the $\zeta$ number is defined by^31^

2 $$\begin{aligned} \zeta =\frac{\Gamma }{2p^{\prime}} \end{aligned}$$

In this expression, $p^{\prime}$ is the effective pitch of the camera sensor when back-imaged by the relay lens having a magnification of *Z*, i.e., $p^{\prime}=p/Z$^31^. This reasoning is for a monochrome camera. For a 2-color camera, one has to consider each array of dexels separately and for one color channel, the nearest neighbor dexels are $p\sqrt{2}$ apart, which has to be considered as the actual pitch of the dexel array. The adapted expression of the $\zeta$ number for a 2-color camera is thus

3 $$\begin{aligned} \zeta =\frac{\Gamma Z}{2\sqrt{2}p}, \end{aligned}$$

which equals the ideal value of 3, in our experimental system.

The QLSI system (Fig. 1b) was implemented on an inverted optical microscope (Fig. 1a), endowed with a 100$\times$ objective lens, 1.3 NA (Olympus UPLFLN100XOP), a tube lens (Thorlabs, TTL200-A) with a focal length of 200 mm instead of 180 mm, which increases the magnification by a factor of 1.11 (the total equivalent magnification of the microscope is thus $100\times 1.11\times 1.193=132$), two light sources: (i) a monochromator (Hypermonochromator, Mountain Photonics GmbH, purchased from Opton Laser International, bandwidth set to 20 nm), set to emit light at $\lambda _{{\text{r}}} = 680$ nm in all experiments, except for the camera characterization where the wavelength was varied from 500 to 780 nm. The monochromator was coupled to an optical fiber illuminating the sample from the top through a Köhler system, the NA of which was set to $\textsf{NA}_\text{i}=0.14$; (ii) A laser beam at $\lambda _\text{b}=488$ nm was illuminating the sample from below to excite the fluorescence of the sample. No emission filter was implemented to cut light from laser illumination because the Bayer filter of the camera already efficiently removed this part of the spectrum.

**Figure 2:**
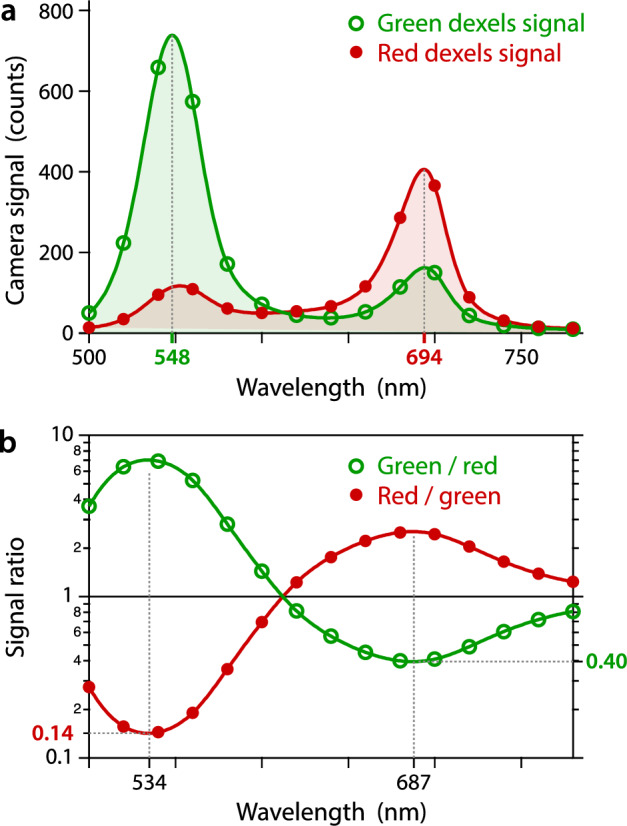
Transmission spectra of the camera. (**a**) Averaged intensity recorded by both color channels of the camera sensor, called Green and Red, as a function of the wavelength (bandwidth 20 nm). (**b**) Ratios of the two spectra displayed in (a), Green/Red and Red/Green.

The Bayer filter of the camera consists of a checkerboard pattern of 2 band-pass dichroic filters centered at 548 nm and 694 nm (Fig. 2a), the former to collect fluorescence and the latter to collect the light from the monochromator and perform the wavefront measurements. The ratio of the detected signals on the two color channels (Fig. 2b) quantifies the cross-talk occurring between neighboring dexels (40% to the green dexels and 14% to the red pixels), an effect that is discussed in the next section.

### Theory and algorithm

**Figure 3:**
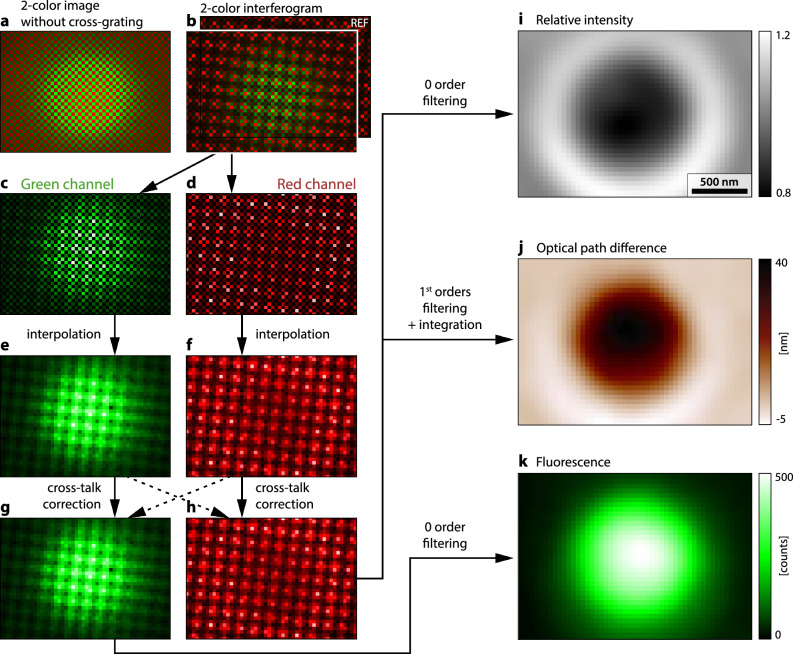
OPD-fluorescence image reconstruction algorithm. (**a**) Raw two-color image detecting both green fluorescence and transmitted red light. (**b**) Same as (a), where the cross-grating has been implemented in the setup. (**c**) Green channel extracted from (b). (**d**) Red channel extracted from (b). (**e**, **f**) Filling of the dark pixels of (c) and (d) by interpolation. (**g**, **h**) Images corrected from the crosstalk. (**i**) Intensity image obtained by demodulation of the red interferogram. (**j**) OPD image obtained by demodulation of the red interferogram (h) around the 1st orders, followed by integration. (**k**) Fluorescence image obtained by demodulation of the green interferogram (g) around the 0 order.

The raw data consists of a 2-color image recorded by the camera (Fig. 3b). The green image is supposed to capture the fluorescence emitted by the sample while the red image collects the light emitted by the monochromator and crossing the sample, containing the information on the OPD of the sample. To process this raw data, we developed an algorithm that is sketched in Fig. 3.

The first step of the algorithm consists of a separation of the two channels (Fig. 3c,d) of the raw colored interferogram (Fig. 3b), leading to images with a checkerboard pattern (half of the pixels of the images are dark because of the Bayer filter). To suppress these dark pixels and obtain images with continuous value variations, bilinear interpolation is performed (Fig. 3e,f), where each dark pixel is simply assigned the average value of the 4 first neighbor pixels. This procedure is common when dealing with color cameras. More sophisticated algorithms can be used, to avoid any blurring effect that could occur when using bi-linear interpolation^33^.

The custom-made color-camera we designed for this study is composed of a monochrome sensor on top of which a Bayer filter was deposited. Such an approach is more flexible and scalable than a monolithic technology in which the Bayer filter is manufactured directly onto the camera sensor (like with common RGB sensors for instance). However, it shows a higher level of inter-dexel leak (called the crosstalk), because of the remaining gap between the Bayer filter and the camera sensor. For applications requiring quantitative measurements, the crosstalk needs to be corrected. Here is the numerical procedure we used to correct crosstalk.

In the absence of crosstalk, the signal detected by each dexel (*i*, *j*) of the camera would be

4 $$\begin{aligned} S_\text{r}(i,j) =&\;\tau \int t_\text{r}(\lambda )Q(\lambda )\Phi (i,j,\lambda )\text{d}\lambda \end{aligned}$$

5 $$\begin{aligned} S_\text{g}(i,j) =&\;\tau \int t_\text{g}(\lambda )Q(\lambda )\Phi (i,j,\lambda )\text{d}\lambda \end{aligned}$$

where the subscripts r and g stand for red and green, $t_\text{r}$ and $t_\text{g}$ are the two transmittance spectra of the Bayer filter, $Q(\lambda )$ is the quantum efficiency of the camera that is wavelength dependent and $\Phi (i, j, \lambda )$ is the number of photons per unit time and wavelength impinging on the dexel (*i*, *j*) (before the Bayer filter), supposed to be constant, at least during the exposure time. The integrals run over the whole light spectrum.

$S_\text{r}$ and $S_\text{g}$ are the images one would ideally measure, theoretically, without crosstalk. However, the images $I_\text{R}$ and $I_\text{G}$ measured in practice suffer from crosstalk, meaning that each dexel is affected by inward and outward leaks from/to the neighboring dexels:

6 $$\begin{aligned} I_\text{r} =&S_\text{r}+\alpha _\mathrm {g\rightarrow r}S_\text{g}-\alpha _\mathrm {r\rightarrow g}S_\text{r}\end{aligned}$$

7 $$\begin{aligned} I_\text{g} =&S_\text{g}+\alpha _\mathrm {r\rightarrow g}S_\text{r}-\alpha _\mathrm {g\rightarrow r}S_\text{g} \end{aligned}$$

where $\alpha _\mathrm {g\rightarrow r}$, resp. $\alpha _\mathrm {r\rightarrow g}$, is the leakage coefficient from green to red dexels (resp. from red to green dexels). In the following, these coefficients will be more simply written $\alpha _\text{g}$ and $\alpha _\text{r}$, respectively. Note that these coefficients are not equal because green and red filters on the Bayer matrix do not have the exact same physical size. Equation (7) can be recast into matrix form

8 $$\begin{aligned} \left[ \begin{array}{c} I_\text{r} \\ I_\text{g} \end{array}\right] = \left[ \begin{array}{cc} 1-\alpha _\text{r} &{} \alpha _\text{g}\\ \alpha _\text{r} &{} 1 - \alpha _\text{g} \end{array}\right] \cdot \left[ \begin{array}{c} S_\text{r} \\ S_\text{g} \end{array}\right] \end{aligned}$$

The true signals, $S_\text{g}$ and $S_\text{r}$, can thus be simply retrieved by a matrix inversion:

9 $$\begin{aligned} \left[ \begin{array}{c} S_\text{r} \\ S_\text{g} \end{array}\right] =&\left[ \begin{array}{cc} 1-\alpha _\text{r} &{} \alpha _\text{g}\\ \alpha _\text{r} &{} 1 - \alpha _\text{g} \end{array}\right] ^{-1} \cdot \left[ \begin{array}{c} I_\text{r} \\ I_\text{g} \end{array}\right] \end{aligned}$$

10 $$\begin{aligned} \left[ \begin{array}{c} S_\text{r} \\ S_\text{g} \end{array}\right] =&\left[ \begin{array}{cc} 1+\beta _\text{r} &{} -\beta _\text{g}\\ -\beta _\text{r} &{} 1 + \beta _\text{g} \end{array}\right] \cdot \left[ \begin{array}{c} I_\text{r} \\ I_\text{g} \end{array}\right] \end{aligned}$$

where the newly defined crosstalk coefficients read

11 $$\begin{aligned} \beta _\text{r}=\frac{\alpha _\text{r}}{1-\alpha _\text{g}-\alpha _\text{r}},\;\;\;\beta _\text{g}=\frac{\alpha _\text{g}}{1-\alpha _\text{g}-\alpha _\text{r}} \end{aligned}$$

We measured them by acquiring two images, without cross-grating, at the two wavelengths of interest: one image, $I_1=\{I_\text{1g},I_\text{1r}\}$, with only a uniform red illumination ($S_\text{g} =0$), at $\lambda _\text{r}=680$ nm, and another image, $I_2=\{I_\text{2g},I_\text{2r}\}$, with only a uniform green illumination ($S_\text{r} =0$) at $\lambda _\text{g}=530$ nm. From Eq. (10), one can write

12 $$\begin{aligned} 0 = (1+\beta _\text{g})*\langle I_\text{1g}\rangle -\beta _\text{r}*\langle I_\text{1r}\rangle \end{aligned}$$

13 $$\begin{aligned} 0 = (1+\beta _\text{r})*\langle I_\text{2r}\rangle -\beta _\text{g}*\langle I_\text{2g}\rangle \end{aligned}$$

giving two equations with two unknowns. The angle brackets mean the average of all the pixels of the image. The solutions of Eqs. (12) and (13) are

14 $$\begin{aligned} \beta _\text{r} =&\frac{\langle I_\text{2g}\rangle \langle I_\text{1g}\rangle + \langle I_\text{2r}\rangle \langle I_\text{1g}\rangle }{\langle I_\text{2g}\rangle \langle I_\text{1r}\rangle - \langle I_\text{1g}\rangle \langle I_\text{2r}\rangle }\end{aligned}$$

15 $$\begin{aligned} \beta _\text{g} =&\frac{\langle I_\text{1r}\rangle \langle I_\text{2r}\rangle +\langle I_\text{1g}\rangle \langle I_\text{2r}\rangle }{\langle I_\text{2g}\rangle \langle I_\text{1r}\rangle - \langle I_\text{1g}\rangle \langle I_\text{2r}\rangle } \end{aligned}$$

To make this procedure accurate, images acquired in the dark must be subtracted to all the images, to cancel the camera offsets (around 20 counts in our case). With our camera, we measured $\beta _\text{r}=0.46$ and $\beta _\text{g}=0.20$.

Once images $S_\text{r}$ and $S_\text{g}$ are obtained using Eq. (10), these interferograms need to be processed. $S_\text{r}$ is processed using the regular CGM algorithm that we depicted in a previous publication^30^, and that we provide on Github^34^. In short, a first demodulation around the 0 order of the Fourier transform of $S_\text{r}$ is performed to retrieve the intensity image, also called the transmittance, *T*. This image is divided by the intensity image $T^\text{ref}$ obtained from the reference interferogram to obtain the relative intensity image $T/T^\text{ref}$ (Fig. 3i). Then, two demodulations are performed by cropping two orthogonal, first-order diffraction spots in the Fourier transform of $S_\text{r}$, leading to two OPD gradient images $\partial _x\delta \ell$, $\partial _y\delta \ell$. From these two orthogonal gradients, the OPD image $\delta \ell ^0$ is retrieved by numerical integration. Here also, as usual in CGM, a reference image $I_\text{r}^\text{ref}$ should be acquired on an empty sample area to compute the reference OPD $\delta \ell ^\text{ref}$ to be subtracted to $\delta \ell$ as a means to cancel beam imperfections, and obtain the final OPD image $\delta \ell =\delta \ell ^0-\delta \ell ^\text{ref}$ (Fig. 3j).

$S_\text{g}$ is processed using the same QLSI algorithm, extracting the 0 order (intensity image)^30,34^ to obtain the fluorescence image $F^0$. Just like with $S_\text{r}$, we also compute the fluorescence (intensity) image of the reference interferogram $F^\text{ref}$. But this time, instead of dividing, we subtract this reference image to get the final fluorescence image, free from the unwanted background $F=F^0-F^\text{ref}$ (Fig. 3k).

The related Matlab algorithm is provided on Github^35^.

**Figure 4:**
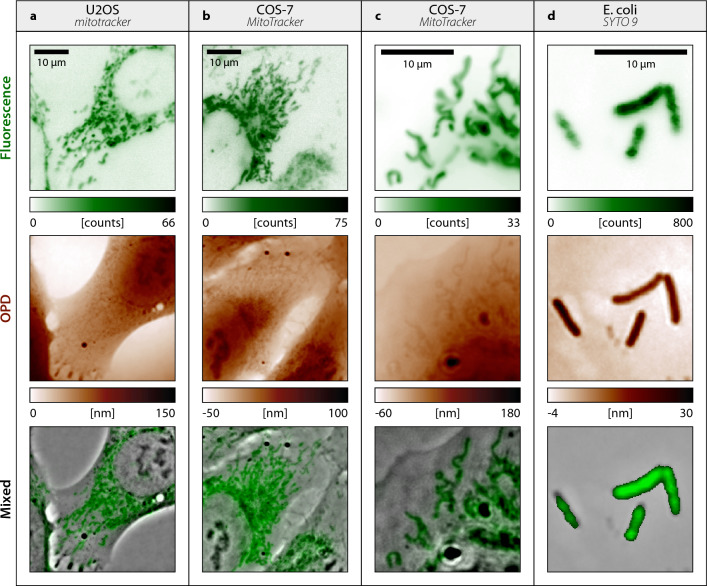
CGM images on various types of live cells. (**a**) U2OS cells, with mitochondria labelled with MitoTracker dyes. Fluorescence and OPD images are displayed, along with a mixed image of the two. (**b**) Same as (**a**), with COS-7 cells. (**c**) Same as (**b**), with a close up on mitochondria. (**d**) Same as (**c**) with Escherichia coli (K12) bacteria, labelled with SYTO 9 stain. Images acquired with $132\times$ magnification, 1.3 NA.

To showcase the versatility of the approach, Fig. 4 presents examples of color-CGM images obtained on various types of cells, namely eukaryotic cells (COS-7, U2OS, both purchased from the American Type Culture Collection) and bacteria (*Escherichia coli*).

These results illustrate the possibility of obtaining simultaneously the global morphology of the cell (via OPD measurements), along with the distribution of specific organelles (here mitochondria), from a single image, and without registration. Note that the high resolution and good sensitivity of CGM also enables the observation of mitochondria on the OPD images themselves, a result reported by Bon et al.^36^.

In the last line of Fig. 4, we show images mixing OPD and fluorescence signals. The OPD image was high-pass filtered, to render the details. The corresponding Matlab algorithm and graphical user interface for mixed image rendering are provided on Github^35^ and described in Supplementary Information.

The next sections focus on different aspects of the technique, which are important to consider in order to obtain correct measurements in terms of accuracy and spatial resolution.

### Chromatic aberrations and numerical refocusing

**Figure 5:**
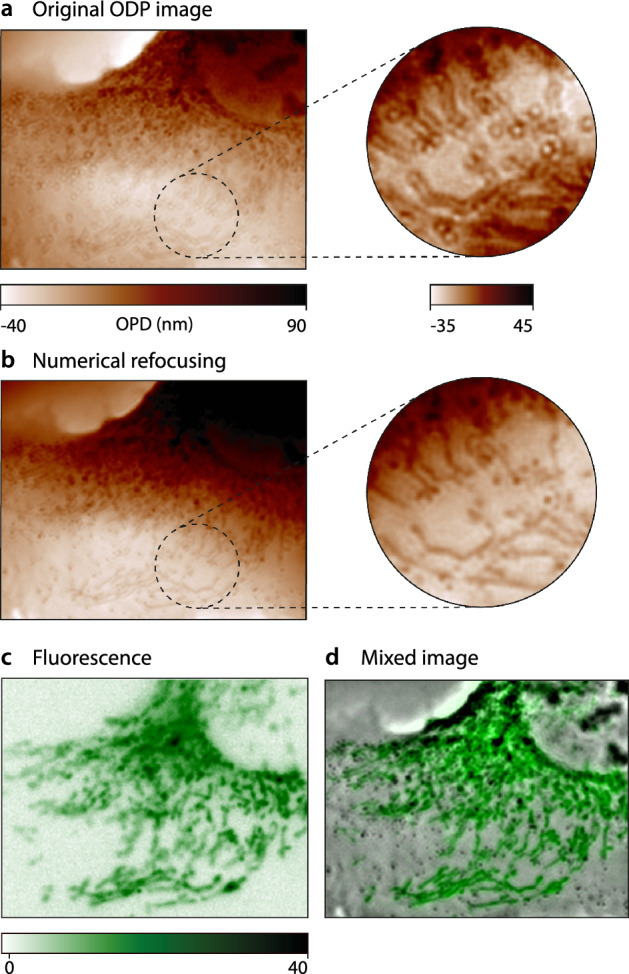
OPD-fluorescence images of COS-7 cells, and focus correction. (**a**) Raw OPD image. The inset highlights the out-of-focus nature of the image. (**b**) Numerically refocused OPD image, from (a). (**c**) Fluorescence image. (**d**) Mixed OPD-fluorescence image.

Figure 5 displays results of OPD/fluorescence images obtained using color-CGM on live COS-7 cells in culture. Cells were labelled using MitoTracker dyes, tagging mitochondria.

The rather distant set of wavelengths used in the experiments, 530 and 680 nm, are likely to yield longitudinal chromatic aberrations, in the sense that the right focus (sample/objective distance) on the red and green channels can be different. In other words, one cannot make the focus simultaneously on the OPD and fluorescence images. Chromatic aberrations come from the objective lens and the relay-lens. The effect is evidenced in the experimental measurements shown in Fig. 5, where the focus was experimentally adjusted on the fluorescence image (Fig. 5c). The zoom inset of Fig. 5a evidences an out-of-focus OPD image, characterized by blurry mitochondria. Fortunately, the occurrence of such an issue can be easily corrected thanks to the unique possibility offered by CGM of numerically refocusing the OPD image, like with any QPM technique. From the knowledge of $\delta \ell$ and *T*, the $\delta \ell$ image at any focus can be retrieved using a Fourier-transform-based light propagation algorithm, which we provide on Github^35^. The result is shown in Fig. 5b, where the inset shows now clearly defined mitochondria.

This common refocusing ability of QPM has a limitation. The numerical refocusing can be performed over a limited distance that depends on the numerical aperture of the illumination $\textsf{NA}_\text{i}$^37^:

16 $$\begin{aligned} D = \frac{\lambda _\text{r}\sqrt{n^2-\textsf{NA}_\text{i}^2}}{\textsf{NA}\times \textsf{NA}_\text{i}} \end{aligned}$$

With a laser illumination, one can achieve $\textsf{NA}_\text{i}\approx 0$ and very good refocusing over arbitrarily large distances. However, CGM enables, and prefers incoherent light sources, because laser illumination tends to create image imperfection such as fringe and speckle, like in DHM. In our case, we use an LED and the Köhler illumination was set to $\textsf{NA}_\text{i}=0.14$. Higher $\textsf{NA}_\text{i}$ values yield better spatial resolution (see further on, Eq. (18)). According to Eq. (16), such an illumination NA offers the possibility to refocus over a typical distance of $D = 1$ µm, which is on the order of what was required experimentally. Please note that Eq. (16) does not strictly define a no-go zone; rather, it provides an estimate of the anticipated operational range. In practice, the focus is adjusted numerically, with close attention paid to small objects, typically vesicles or the lamellipodium edge, to make them as sharp as possible. With our microscope, the focus difference between OPD and fluorescence images ranges from 0.75 to 1 µm. Figure 5b displays the result of a refocusing procedure, and the proper reconstruction of mitochondria in focus, where the numerical refocusing distance was set to 0.95 µm.

### Influence of the grating position on the measurement accuracy

**Figure 6:**
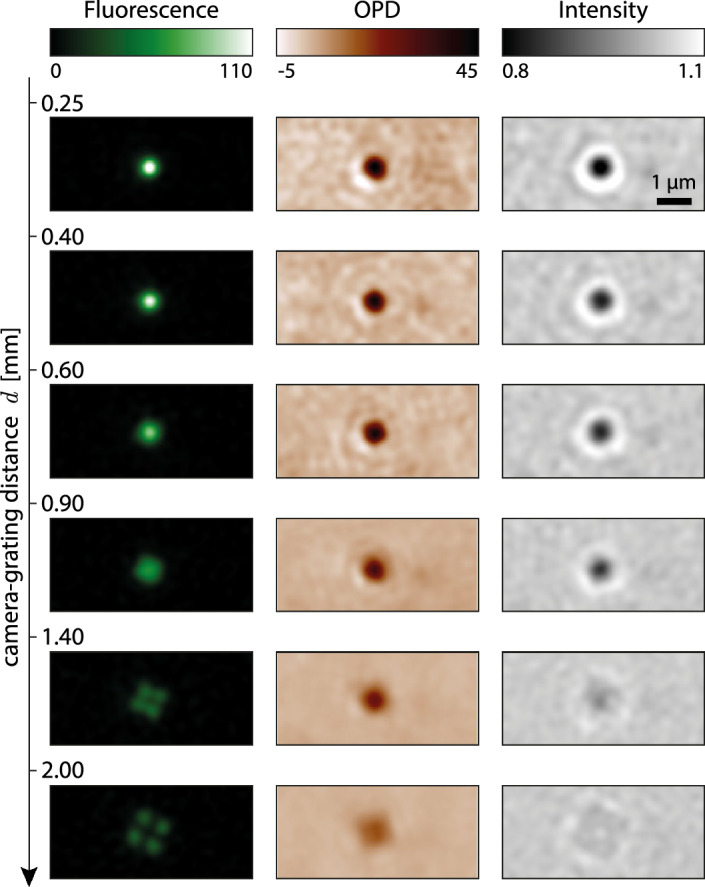
Color-CGM images of a 500-nm polystyrene bead as a function of the camera-grating distance *d*, from $d=0.25$ to 2 mm. The bead is in water, lying on glass, imaged with a 132$\times$ magnification, 1.3 NA.

**Figure 7:**
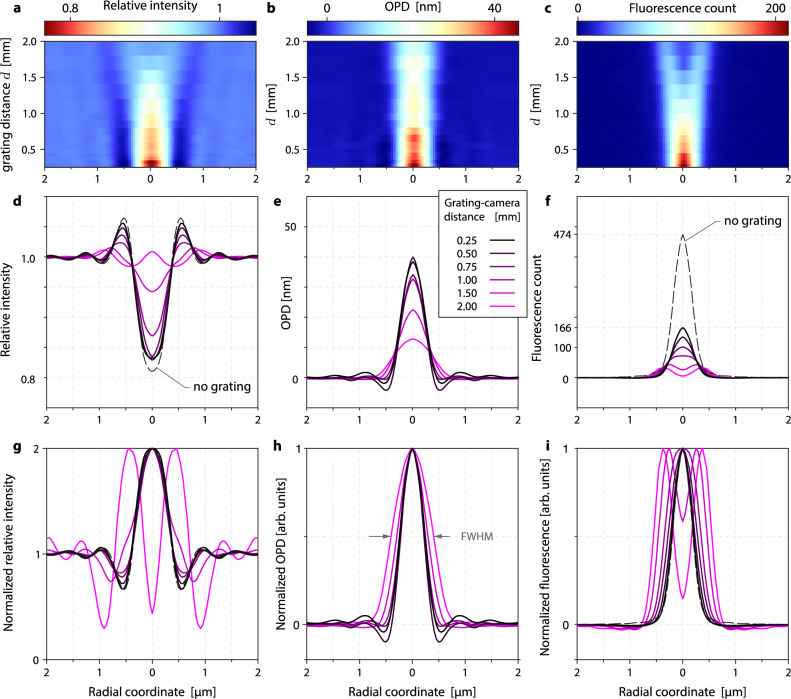
Influence of the grating-camera distance on the image quality, illustrated on a 500-nm polystyrene bead, imaged by color-CGM with 132$\times$ magnification, 1.3 NA. (**a**, **b**, **c**) Maps of radial cross-cuts vs grating distance, for (**a**) intensity, (**b**) OPD and (**c**) fluorescence. (**d**, **e**, **f**) Plots of radial cross-cuts vs 6 different grating distances, for (**d**) intensity, (**e**) OPD and (**f**) fluorescence. (**g**, **h**, **i**) Same as (**d**, **e**, **f**), but normalized to unity.

In CGM, the QLSI device offers one degree of freedom, the camera-grating distance *d*, usually in the millimeter range. The further the grating, the better the signal to noise ratio. However, if the grating is moved too far away, four-fold symmetry artifacts appear on the image^31^. There exists thus a tradeoff in CGM between precision and accuracy.

Figure 6 illustrates this tradeoff by showing fluorescence, OPD and intensity images of a 500-nm fluorescent bead acquired using color-CGM. At small camera-grating distance, the noise is noticeable, but the images contrast is good. When the grating is moved away, the noise amplitude looks damped, but the bead image is spreading, until an artifactual 4-fold symmetry appears, in relation with the 4-fold symmetry of the QLSI grating. In practice, the grating-camera distance must be adjusted, keeping in mind this trade-off between accuracy and signal to noise ratio. Calibration can be done using fluorescent beads as shown in Fig. 6. In this study, we chose $d=0.91$ mm^31^.

Figure 7 presents results of a detailed study of the influence of the camera-grating distance. Figures 7a–c plot the radial profiles of experimental color-CGM images as a function of the grating position *d*, showing the spreading and the damping of the signal. Figures 7d–f plot the same data as in a–c, but only for a set of 6 values of *d*. In Figs. 7d,f, the measured intensity and fluorescence profiles *without grating* are also plotted. One can see that the implementation of the grating does not affect the relative intensity profile for small *d* values, as expected. However, the fluorescence intensity is damped, down to 35%, once the QLSI grating is placed (Fig. 7f), because of the averaged transmittance of the grating that is theoretically 4/9 = 44% (because of the opaque lines of the cross-grating), and because of the light sent on other diffraction orders than the 0 order. This reduced light transmission tends to increase the exposure time and consequently any photobleaching, by a least a factor of 3. Movie 1 in Suppl. Info. shows mitochondria actively moving in COS7 cells, in fluorescence and OPD, where some photobleaching can be observed.

### Spatial resolution in color-CGM

Two spatial resolutions enter into play in this study, one related to the OPD/intensity images, and one related to the fluorescence image. They are different first because the wavelengths are different, and second because the spatial resolution depends on the imaging modality. For self-luminous object (here with fluorescence microscopy), the spatial resolution is given by

17 $$\begin{aligned} r_\text{g}=\frac{\lambda _\text{g}}{2\textsf{NA}} \end{aligned}$$

In the case of wide-field microscopy, like in CGM, the NA of the illumination $\textsf{NA}_\text{i}$ also matters, and the spatial resolution is given by

18 $$\begin{aligned} r_\text{r}=\frac{\lambda _\text{r}}{\textsf{NA}+\textsf{NA}_\text{i}} \end{aligned}$$

In our experiments, where $\textsf{NA}=1.3$, $\textsf{NA}_\text{i}=0.14$, $\lambda _\text{g}=548$ nm and $\lambda _\text{r}=680$ nm, one obtains $r_\text{r} = 472$ nm and $r_\text{g}=257$ nm.

To reach these theoretical spatial resolutions in practice, the microscope must be able to capture a sufficiently large range of spatial frequencies. In regular optical microscopy, the dexel size *p* is the parameter to be considered to ensure the capture of the highest spatial frequencies of the image. *p* has to be small enough according to the Nyquist criterion. In CGM, the reasoning is slightly different. The dexel size has to be adjusted, not in relation with the image, but in the relation with the grating, because the role of the camera, in CGM, is to sample the bright spots of the interferogram: each bright spot of the grating has to be sampled by 3 dexels in lateral size ($\Gamma /2=3p$). In CGM, to reach the theoretical diffraction limits dictated by Eqs. (17) and (18), the parameter of interest if rather the size of the grating unit cell (the grexel^31^). Each grexel produces 4 spots on the camera, of half the size of the grexel, $\sqrt{2}\Gamma /2$. The image is sampled by this array of spots, with a periodicity that is three times smaller than the dexel periodicity. Consequently, CGM yields a reduction of the pixel number of the image, compared with regular imaging without grating. This is why a reduction in image *definition* may lead to a reduction in image *resolution* a priori, but not necessarily: if the unit cell of the grating (and consequently the dexel size of the camera) is small enough to sufficiently oversample the image, then the reduction in definition does not reject any spatial frequency of the image. According to the Nyquist criteria, this relation must be satisfied to reach the diffraction limit:

19 $$\begin{aligned} r_\text{r}=\frac{\lambda _\text{r}}{\textsf{NA}+\textsf{NA}_\text{i}}\ge \frac{\Gamma \sqrt{2}}{M}=r_\text{lim} \end{aligned}$$

**Figure 8:**
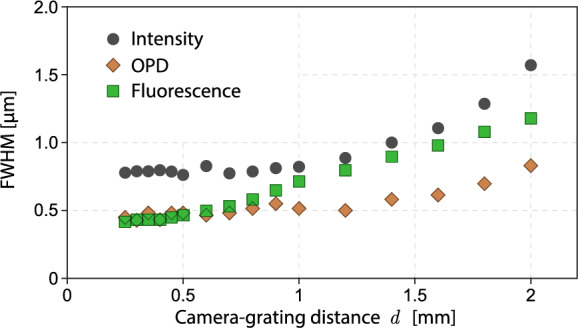
FWHM of the bead image as a function of the camera-grating distance, for the three types of images, namely intensity, OPD and fluorescence.

Let us apply this rule to the set up configuration we used in this work, where $\lambda =680$ nm, $\textsf{NA}=1.3$, $\textsf{NA}_\text{i}=0.14$, and $M=111$. It gives a theoretical spatial resolution of 472 nm, and a limiting spatial resolution of 495 nm. Hence, the inequality (19) is quasi satisfied, meaning that the grating period is such that it captures the highest frequencies of the image, and barely degrades the spatial resolution of OPD images. In other words, our setup configuration reaches the diffraction limit in OPD.

However, the spatial resolution in fluorescence, albeit acceptable for the systems under study, is supposed to be affected, because $r_\text{g}<r_\text{lim}$. According to Eq. (19) , this problem could be circumvented by increasing the magnification *M* of the microscope before the image reaches the grating. In other words, one can increase the magnification of the microscope (objective, tube lens), but playing with the magnification *Z* of the relay lens is not effective. Still according to Eq. (19), one can also use a cross-grating with a smaller unit cell, provided the dexel size of the camera, or the magnification of the relay lens *Z* are adjusted consistently to ensure a $\zeta$ number of 3 (Eq. (3)).

Importantly, in CGM, the spatial resolution can also be affected by the camera-grating distance. The closer the grating, the better the spatial resolution. This rule is illustrated by the measurements presented in Fig. 7g–i. These graphs represent the same crosscuts as in Figs. 7d–f, but normalized by their maximum value to better compare their width, and give information on the loss of spatial resolution. One can see that for small camera-grating distances, the radial crosscuts of the intensity and fluorescence images remain identical to the crosscuts measured without grating, indicating that the spatial resolution is preserved, and confirming the discussion following Eq. (19). However, when the grating is moved away from the camera, the profiles markedly spread. To quantify the loss of spatial resolution when the grating is moved away, the full-width half maximum (FWHM) of all the intensity, OPD and fluorescence images have been measured, and the results are displayed in Fig. 8. The FWHM naturally increases when moving away the grating, but remains quite constant below 0.6 mm, which can be considered as the safety range to preserve the spatial resolutions on all the images.

## Discussion

Coupling fluorescence and phase/wavefront microscopy is gaining interest because it enables the monitoring of cell morphology, biomass transport and quantitative growth rate in parallel with fluorescence monitoring of cell activity. This phase/fluorescence or wavefront/fluorescence association is unfortunately not popularized yet in the bioimaging community, because of the difficulty of implementing, on the same microscope, phase/wavefront imaging with fluorescence imaging. Color-CGM is the simplest, yet very effective way to accomplish this task, because it simply consists in plugging a camera-like system on a microscope.

The envisioned applications are numerous. Among others, color-CGM could avoid the use of DAPI labelling, because nuclei are clearly visible on OPD images.

One can also imagine that color-CGM could benefit studies on lamellipodium dynamics, perfectly visible on OPD images, and correlate them with fluorescence images of the cytoskeleton for instance.

Acquiring both OPD and fluorescence images on bacteria is relevant in cases where not all the bacteria are supposed to emit fluorescence. For instance, when conducting live/dead viability measurements, one usually has to use two tags, one tagging live cells, the other dead cells, making the approach complex. Using color-CGM, one can just use a single tag, and a single image acquisition. The localization of all the bacteria would be given by the OPD images, and only the live bacteria would appear fluorescent. Also, fluorescent labelling can be used to differentiate between distinct bacterial species in a mixed population, one being fluorescent, the other not.

A perfect spatial overlap of OPD and fluorescence images, as permitted by color-CGM, is of utmost importance for small objects, like bacteria or cell organelles. A small imprecision in the registration of fluorescence and OPD images would place the fluorescent signal in the wrong place of a bacteria. Because the spatial match is perfect in color-CGM by nature, such a problem is avoided. In the same spirit, an extremely good spatial accuracy is necessary for any study related to super-resolution microscopy. Color-CGM could be used in super-resolution microscopy, like STORM or PALM, and give an unprecedented map of super-localized molecules in cells, along with the overall cell morphology.

The technique could be further improved. First, the camera we used (10-bit, $\sim 50$% quantum efficiency, not cooled) does not meet the standard of fluorescence microscopy. There are more efficient cameras that would give rise to less photobleaching and image noise. Then, we are using a relay-lens because we wanted to vary the camera-grating distance, but nothing prevents from actually fixing the grating at 0.6 mm from the camera sensor. It would lead to a more compact, portable system, with less chromatic aberrations, and more likely to be commercialized. The two color channels of the exact same camera could also be used to conduct dual-color OPD imaging. Finally, the number of dexel colors is not limited to green and red in general. We stick to this configuration in this article for the sake of simplicity, and because green is the most commonly investigated range of fluorescence emission. However, cameras with other fluorescence wavelength ranges could be imagined, or even with several fluorescence wavelength ranges using 3-color or 4-color cameras. In this latter case, the image definition will be reduced by a factor of $\sqrt{2}$ because the dexels of same color will naturally be further apart compared with the 2-color Bayer filter we used for this study.

## Editorial policies for:

Springer journals and proceedings: https://www.springer.com/gp/editorial-policies

Nature Portfolio journals: https://www.nature.com/nature-research/editorial-policies

*Scientific Reports*: https://www.nature.com/srep/journal-policies/editorial-policies

BMC journals: https://www.biomedcentral.com/getpublished/editorial-policies

### Supplementary Information

Supplementary Information 1.

Supplementary Video 2.

## Data Availability

All data required to reproduce the results can be obtained from the corresponding author upon a reasonable request.

## Code availability

All the data were processed using Matlab. All the Matlab codes required to process the data of the article are provided on these Github repositories:

https://github.com/baffou/CGMprocess, to process the interferograms into OPD and intensity images

https://github.com/baffou/colorCGM, an add-on to the previous repository, to process colored-interferograms into OPD, intensity and fluorescence images, and to mix the OPD and fluorescence images into single color images.
