# Author Correction: Single-shot quantitative phase-fluorescence imaging using cross-grating wavefront microscopy

**DOI:** 10.1038/s41598-024-61183-3

**Published:** 2024-05-08

**Authors:** Baptiste Marthy, Maëlle Bénéfice, Guillaume Baffou

**Affiliations:** Institut Fresnel, CNRS, Aix Marseille Univ, Centrale Med, Marseille, France

Correction to: *Scientific Reports* 10.1038/s41598-024-52510-9, published online 25 January 2024

The original version of this Article contained errors.

In Figure 4, the OPD scale bar was incorrect in panel (a). The original Figure 4 and accompanying legend appear below.

**Figure 4:**
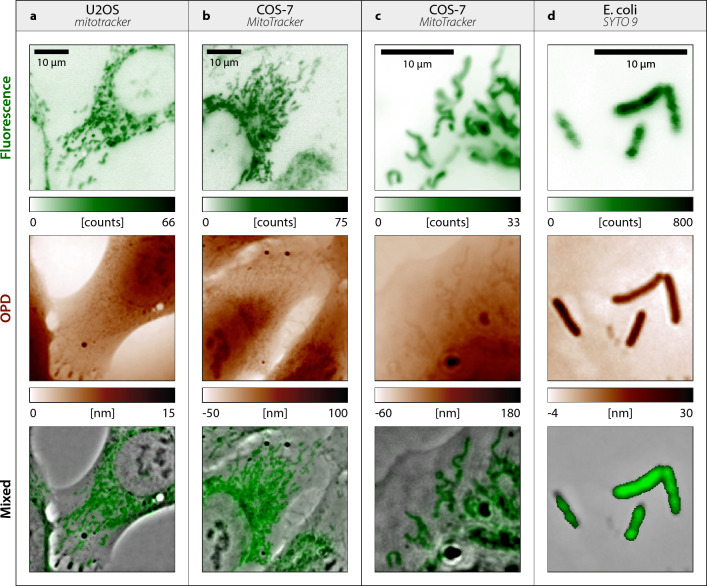
CGM images on various types of live cells. (**a**) U2OS cells, with mitochondria labelled with MitoTracker dyes. Fluorescence and OPD images are displayed, along with a mixed image of the two. (**b**) Same as (**a**), with COS-7 cells. (**c**) Same as (**b**), with a close up on mitochondria. (**d**) Same as (**c**) with Escherichia coli (K12) bacteria, labelled with SYTO 9 stain. Images acquired with $132\times$ magnification, 1.3 NA.

In addition, the Competing interests statement in this paper was incomplete. It read

“The authors declare no competing interests.”

and now reads

“G. Baffou collaborated with SILIOS Technologies as a consultant for the development of a wavefront camera unrelated to this study. SILIOS Technologies were not involved with the design or execution of this project, nor had any input into its outcomes. The other authors declare no competing interests.”

Finally, due to G. Baffou’s collaboration with SILIOS Technologies, J. Savatier wishes to have his name removed from the Acknowledgements section, which previously read

“The authors thank J. Savatier and S. Massieau for helping on the eukaryotic cell culture, and L. F. Wu for providing the *E. coli* bacteria.”

and now reads

“The authors thank S. Massieau for helping on the eukaryotic cell culture, and L. F. Wu for providing the *E. coli* bacteria.”

The original Article has been corrected.

