# Supplementary material for: Single-shot quantitative phase-fluorescence imaging using cross-grating wavefront microscopy
Source: Sci Rep. 2024 Jan 25;14:2142. doi: 10.1038/s41598-024-52510-9 (PMC10810858; doi:10.1038/s41598-024-52510-9)
Supplement: Supplementary file 1 — Supplementary Information 1. [file 41598_2024_52510_MOESM1_ESM.pdf]

Supplementary Information

Single-shot quantitative phase-fluorescence imaging using cross-grating wavefront microscopy

Baptiste Marthy[1], Maëlle Bénéfice[1], and Guillaume Baffou[1,*]

[1] *Institut Fresnel, CNRS, Aix-Marseille Université, Centrale Marseille, Marseille, France*
[*] *guillaume.baffou@fresnel.fr*

1. COMPARATIVE TABLE OF THE DIFFERENT OPD-FLUORESCENCE CORRELATION TECHNIQUES

Table reviewing the experimental approaches to combine fluorescence with quantitative phase/wavefront imaging, and their drawbacks, namely 2-camera systems [1–6], 1 single camera with the two images projected on separate regions of interest of the interest (possible but not implemented yet by the community in the frame of fluorescence-OPD correlation imaging), and sequential acquisitio with a single camera, by the motion of a mechanical element [7].

	delay between images	need for registration	cameras synchronisation
2-camera systems	no	yes	yes
1 camera, 2 ROI	no	yes	no
sequential acquisition	yes	no	no
color-CGM	no	no	no

Fig. S1. Comparative table of the different OPD-fluorescence correlation techniques

2. DETERMINATION OF THE FWHM OF BEAD IMAGES

Figure 8 of the main manuscript plots what we called the full-width half-maximum (FWHM) measured on the images of 500-nm beads, in intensity, OPD and fluorescence. On fluorescence and OPD images, we measured the actual FWHM.

However, on the intensity images, there were so many bounces in the Airy pattern that taking the actual FWHM would not have led to an actual estimation of the spreading of the image. For this reason, we rather consider an integral calculation to estimate the spreading of the image, according to the following expression:

$$s = 2.355\sqrt{\frac{\int x^2 f(x)\mathrm{d}x}{\int f(x)\mathrm{d}x}} \quad \textbf{(1)}$$

f is the radial profile of the image. The pre-factor is here to make this expression equal the FWHM, when applied to a Gaussian shape.

3. DESCRIPTION OF THE MATLAB CODE FOR TREATING THE RAW 2-COLOR INTERFEROGRAM

On our Github account [8], we provide a Matlab package, `baffou/CGMprocess`, suited to process the interferograms into intensity and OPD images. This package was developed in the frame of a previous publication [9].

In the frame of this publication, we created a new repository, `baffou/colorCGM`, on the same Github account [10], suited to process 2-color interferograms into intensity, OPD and fluorescence images. It is written as an add-on to the `baffou/CGMprocess` repository.

4. DESCRIPTION OF THE GRAPHICAL USER INTERFACE (GUI) FOR RENDERING TWO-COLOR OPD-FLUORESCENCE IMAGES

The Github repository `baffou/colorCGM` also contains a Matlab app, `colorCGMdisplay.mlapp`, suited to render 2-color OPD-fluorescence images, as the ones displayed in Figs. 4 and 5d of the main manuscript.

The gui is endowed with 4 tabs. The first is to be used to import the OPD and fluorescence images (must be ascii files). The load button imports the images and creates the composite image. The second tab is aimed to adjust the color scales on the original fluorescence and OPD images, just for a better visualization of these two images, if necessary. The third tab is the important tab. It offers a set of parameters that need to be adjusted to control the rendering of the mixed image. The min and max

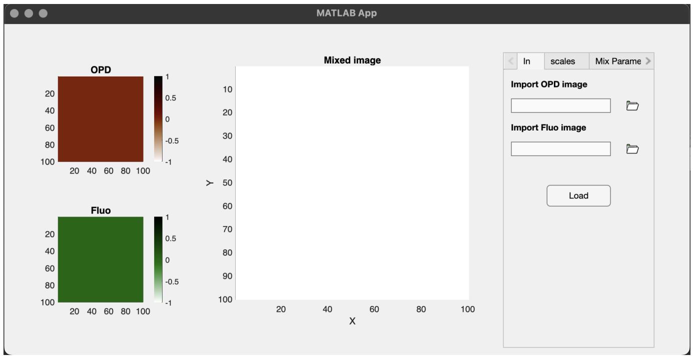

Fig. S2. Screenshot of the main window of the Matlab app.

values sets the images limits. The high pass value removes the low frequencies of the OPD images. The threshold value affects the extension of the fluorescence image. Finally, the fourth tab exports an eps file containing the two original images, with the mixed image, taking into account all the parameters set in tabs 2 and 3.

5. MOVIE M1 OF OPD FLUORESCENCE IMAGING

Movie `M1_COS7.avi` is a sequence of OPD-fluorescence images obtained by CGM on COS-7 cells labelled with MitoTracker dyes, using the same microscope configuration as in Fig. 4 and 5.

REFERENCES

1. K. Wen, Z. Gao, R. Liu, Y. Ma, J. J. Zheng, S. An, T. Kozacki, and P. Gao, "Structured illumination phase and fluorescence microscopy for bioimaging," Appl. Opt. **62**, 4871–4879 (2023).
2. S. Chowdhury, W. J. Eldridge, A. Wax, and J. A. Izatt, "Structured illumination multimodal 3d-resolved quantitative phase and fluorescence sub-diffraction microscopy," Biomed. Opt. Express **8**, 2496–2518 (2017).
3. D. Dong, X. Huang, L. Li, Y. Mo, G. Zhang, Z. Zhang, J. Shen, W. Liu, Z. Wu, G. Liu, Y. Liu, H. Yang, Q. Gong, K. Shi, and L. Chen, "Super-resolution fluorescence-assisted diffraction computational tomography reveals the three-dimensional landscape of the cellular organelle interactome," Light. Sci. Appl. **9**, 11 (2020).
4. S. Shin, D. Kim, K. Kim, and Y. K. Park, "Super-resolution three-dimensional fluorescence and optical diffraction tomography of live cells using structured illumination generated by a digital micromirror device," Sci. Rep. **8**, 9183 (2018).
5. P. Bon, J. Savatier, M. Merlin, B. Wattellier, and S. Monneret, "Optical detection and measurement of living cell morphometric features with single-shot quantitative phase microscopy," J. Biomed. Opt. **17**, 076004 (2012).
6. D. Alonso, J. Garcia, and V. Micó, "Fluholoscopy—compact and simple platform combining fluorescence and holographic microscopy," Biosensors. **13**, 253 (2023).
7. Y. K. Park, T. Yamauchi, W. Choi, R. Dasari, and M. S. Feld, "Spectroscopic phase microscopy for quantifying hemoglobin concentrations in intact red blood cells," Opt. Lett. **34**, 3668–3670 (2009).
8. G. Baffou, "https://github.com/baffou/cgmprocess," .
9. G. Baffou, "Quantitative phase microscopy using quadriwave lateral shearing interferometry (qlsi): principle, terminology, algorithm and grating shadow description," J. Phys. D: Appl. Phys. **54**, 294002 (2021).
10. G. Baffou, "https://github.com/baffou/colorcgm," .
